# A standardized set of 3-D objects for virtual reality research and applications

**DOI:** 10.3758/s13428-017-0925-3

**Published:** 2017-06-23

**Authors:** David Peeters

**Affiliations:** Max Planck Institute for Psycholinguistics, P.O. Box 310, NL-6500 AH Nijmegen, The Netherlands

**Keywords:** Virtual reality, 3D-objects, Database, Stimuli

## Abstract

The use of immersive virtual reality as a research tool is rapidly increasing in numerous scientific disciplines. By combining ecological validity with strict experimental control, immersive virtual reality provides the potential to develop and test scientific theories in rich environments that closely resemble everyday settings. This article introduces the first standardized database of colored three-dimensional (3-D) objects that can be used in virtual reality and augmented reality research and applications. The 147 objects have been normed for name agreement, image agreement, familiarity, visual complexity, and corresponding lexical characteristics of the modal object names. The availability of standardized 3-D objects for virtual reality research is important, because reaching valid theoretical conclusions hinges critically on the use of well-controlled experimental stimuli. Sharing standardized 3-D objects across different virtual reality labs will allow for science to move forward more quickly.

Visual representations of individual objects have been an essential type of experimental stimulus in several domains of scientific inquiry including attention, language, memory, and visual perception research. Already at the end of the 19th century, James McKeen Cattell developed an ingenious instrument that allowed for the consecutive presentation of individual pictures of objects (and other visual stimuli such as words and numerals) to an observer (Cattell, 1885). The use of visual stimuli in such an experimental context led to theoretically interesting findings such as that words are named faster than pictures and that pictures are named faster in one’s first language than in one’s second language (Cattell, 1885; Levelt, 2013). Over the years, picture-naming tasks have continued to play a pivotal role in psychological and neurological research—for instance, in the development of cognitive models of speech production (e.g., Levelt, Roelofs, & Meyer, 1999).

Reaching meaningful and valid theoretical conclusions critically hinges on the use of well-controlled experimental stimuli. Therefore, standardized, normative databases of picture stimuli have been crucial in controlling for the factors that influence picture recognition and picture-naming latencies, as well as in enabling the comparison of results across different studies and different samples of participants. The most influential standardized picture database to date was developed by Snodgrass and Vanderwart (1980). It consists of 260 black-and-white line drawings standardized for name agreement (the degree to which participants produce the same name for a given picture), image agreement (the degree to which participants’ mental image of a concept corresponds to the visually depicted concept), familiarity (the degree to which participants come in contact with or think about a depicted concept in everyday life), and visual complexity (the amount of detail or intricacy of line in the picture) in native speakers of American English. Over the years, similar picture databases have been introduced and standardized for other languages, including British English, Bulgarian, Dutch, French, German, Hungarian, Icelandic, Italian, Japanese, Mandarin Chinese, and Modern Greek (Alario & Ferrand, 1999; Barry, Morrison, & Ellis, 1997; Bonin, Peereman, Malardier, Méot, & Chalard, 2003; Dell’Acqua, Lotto, & Job, 2000; Dimitropoulou, Duñabeitia, Blitsas, & Carreiras, 2009; Martein, 1995; Nishimoto, Miyawaki, Ueda, Une, & Takahashi, 2005; Nisi, Longoni, & Snodgrass, 2000; Sanfeliu & Fernandez, 1996; Szekely et al., 2004; Van Schagen, Tamsma, Bruggemann, Jackson, & Michon, 1983; Viggiano, Vannucci, & Righi, 2004; Vitkovitch & Tyrrell, 1995; Wang, 1997).

Such black-and-white line drawings typically used in experiments are abstractions of real-world objects. They lack the texture, color, and shading information of the natural objects that we encounter in the real world. One may therefore doubt whether results obtained in studies using line drawings will fully generalize to everyday situations. In a first attempt to increase the ecological validity of experimental stimuli, standardized databases have been developed that include grayscale or colored photographs of objects (e.g., Adlington, Laws, & Gale, 2009; Brodeur, Dionne-Dostie, Montreuil, & Lepage, 2010; Migo, Montaldi, & Mayes, 2013; Moreno-Martínez & Montoro, 2012; Viggiano et al., 2004). Indeed, in certain cases color information in a picture or a line drawing enhances object recognition, such as when several objects within a category (e.g., types of fruit) have relatively similar shapes (e.g., apple, orange, peach) but different diagnostic colors (see, e.g., Laws & Hunter, 2006; Price & Humphreys, 1989; Rossion & Pourtois, 2004; Wurm, Legge, Isenberg, & Luebker, 1993). Importantly, the use of more ecologically valid stimuli significantly increases the odds of experimental findings being generalizable to everyday situations of object recognition, naming, and memory. Despite the availability of color and surface details in photographs of objects, there is still a large gap between observing a picture of an object on a small computer monitor in the lab and encountering that object in the real world. One important difference is the two-dimensional (2-D) nature of the line drawing or photograph versus the three-dimensional (3-D) nature of the objects we encounter in the wild.

In further pursuit of establishing the ecological validity of psychological and neuroscientific findings and theory in general, researchers have now started to exploit recent advances in immersive virtual reality (VR) technology (see Bohil, Alicea, & Biocca, 2011; Fox, Arena, & Bailenson, 2009; Peeters & Dijkstra, 2017; Slater, 2014). In immersive virtual environments, participants’ movements are tracked and their digital surroundings rendered, usually via large projection screens or head-mounted displays (Fox et al., 2009). This allows researchers to immerse participants in rich environments that resemble real-world settings, while maintaining full experimental control. Critically, such environments will often contain a multitude of 3-D objects. One can think of the furniture in a virtual classroom, the food items in a virtual restaurant, the groceries in a virtual supermarket, or even the clothes that a virtual agent or avatar is wearing. Whether participants recognize the 3-D objects will depend, among other factors, on those objects’ graphical quality. However, producing realistic 3-D objects takes time as well as graphic design skills. An open-access database of standardized 3-D objects for VR experiments and applications would be an important step forward in facilitating such research and making the findings comparable across different studies and different groups of participants.

The present study, therefore, introduces a database of 147 colored 3-D objects standardized for name agreement, image agreement, familiarity, visual complexity, and corresponding lexical characteristics of the modal object names. The 3-D objects are freely available from an online database and can be used for VR and augmented reality research and applications. Researchers may use them in the virtual, 3-D equivalents of traditional object recognition and object-naming experiments, to test whether original findings will generalize to situations of more naturalistic vision that include depth cues and richer environments (e.g., Eichert, Peeters, & Hagoort, 2017; Tromp, Peeters, Meyer, & Hagoort, 2017). Moreover, these 3-D objects can be used in any virtual setting that requires the presence of objects. Using a 3-D object from the database will be faster than designing the object from scratch. Moreover, on the basis of the standardized norms, researchers may select 3-D objects that fit the purpose of their specific research question.

## Method

### Participants

A total of 168 native Dutch speakers (84 female, 84 male; mean age 22 years old; age range 18–31 years) participated in the study. Each task (name agreement, image agreement, familiarity, and visual complexity) included 42 different participants (21 female, 21 male). One additional participant in the name agreement task was replaced due to technical problems during the experiment. All of the participants were Dutch; studied in Nijmegen, The Netherlands; and had Dutch as their single native language. They were university students, which means that they had been enrolled in at least 12 years of formal education. They all had normal or corrected-to-normal vision and no language or hearing impairments or history of neurological disease. The participants provided informed consent and were paid for participation. Ethical approval for the study was granted by the ethics board of Radboud University’s Faculty of Social Sciences.

### Materials

**Fig. 1:**
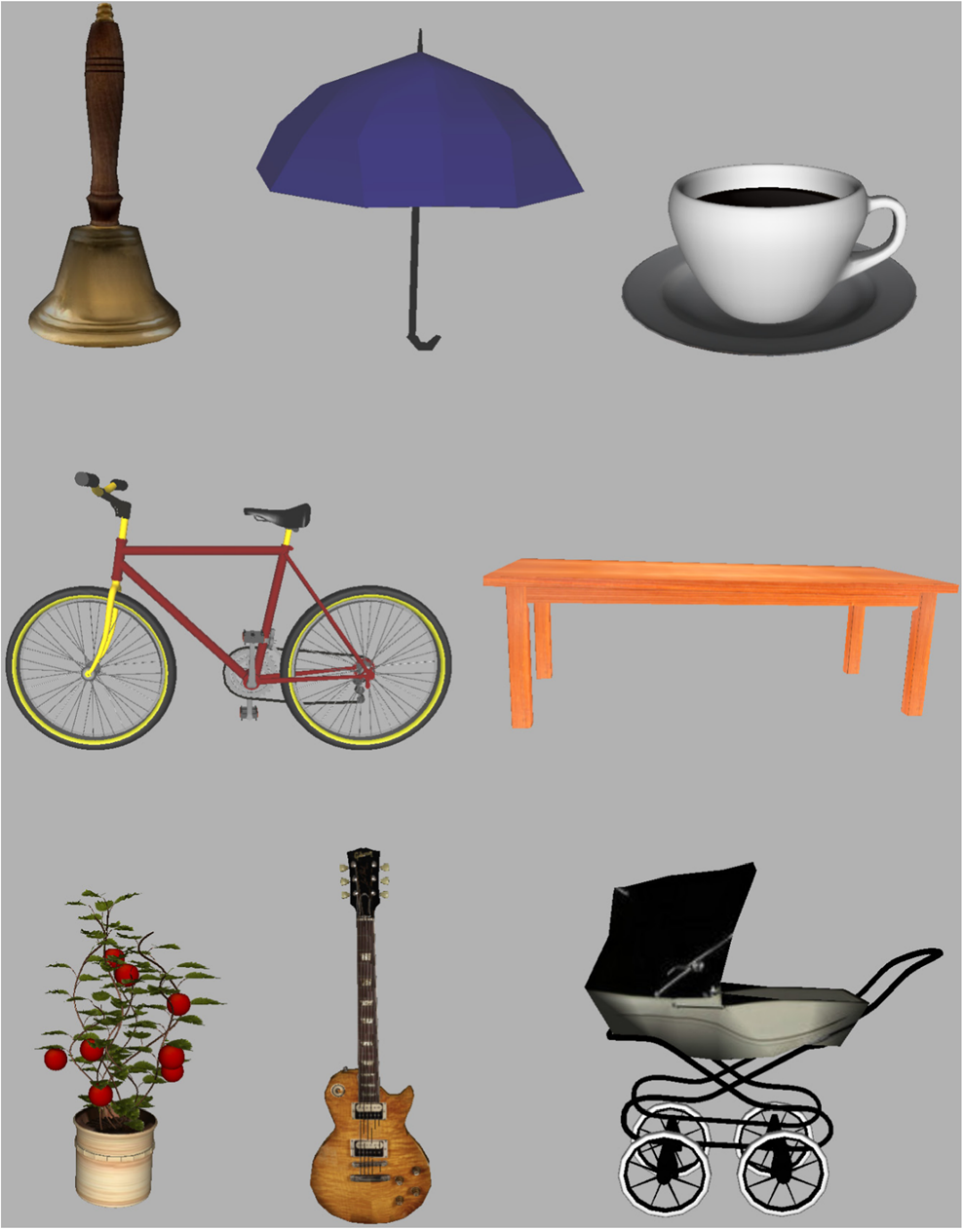
Screenshots of a subset of the standardized 3-D objects

A total of 150 3-D objects were created by an in-house graphics designer for ongoing experimental VR studies in our lab. The objects were created for immersive virtual environments using the 3-D computer graphics software Autodesk Maya (Autodesk Inc., 2016). Each object was designed to represent a stereotypic instance of a specific object name. The objects belonged to several different semantic categories, including food items, furniture, clothing, toys, and vehicles (see the URL provided below and Fig. 1). The texture added to the objects’ surfaces was either custom-made in the graphics software or taken from freely available pictures from the Internet. Three objects were not included in the database, because the majority of participants in the name agreement task did not recognize the object intended by the designer. Hence, the database contains 147 objects in total. All of these objects are made freely available from an online source in both .OSGB and .FBX format, such that they can be used with the Vizard or Unity 3D software. For each object, an.OSGB file, an.fbx file, and a 2-D screenshot are provided at https://hdl.handle.net/1839/CA8BDA0E-B042-417F-8661-8810B57E6732@view. Figure 1 presents screenshots of a subset of the objects (in 2-D).

### Procedure

In each task, after having provided informed consent, participants were seated in a chair in the middle of a CAVE system (Cruz-Neira, Sandin, & DeFanti, 1993), such that the three screens covered their entire horizontal visual field (see below). They put on VR glasses, which were part of a tracking-system that monitored the position and direction of the participant’s head, controlling the correct perspective of the visual display. The eyes of the participant were approximately 180 cm away from the middle screen. Objects were presented one by one in random order against a simple background for 7 s on the center of the screen facing the participant. We aimed to present the objects in expected real-world size. A number (1 to 150) was displayed next to the object that corresponded with a number on the answer sheet or file. The procedure in each of the four tasks was kept similar to the procedure used for standardization of picture databases (e.g., Snodgrass & Vanderwart, 1980). For all four tasks, participants were informed that we were setting up a database of 3-D objects made by an in-house designer and that we would like to know people’s opinion about the objects. Each task consisted of a single session without breaks. To include as many objects as possible in the database, no practice session with practice objects preceded the task. Instead, the experimenter checked before the start of the experiment whether the participant completely understood the task. For these simple tasks, this procedure worked well.

In the *name agreement* task, participants were instructed to carefully look at the object and type the name of each object into a laptop they held on their lap. They were told that a name could consist of a maximum of two words. They were asked to type in “OO” (for *Object Onbekend*, “unknown object”) if they did not know the object, “NO” (for *Naam Onbekend*, “name unknown”) if they knew the object but not its name, and “PT” (for *Puntje van de Tong*, “tip of the tongue”) for objects that elicited a tip-of-the-tongue state. Henceforth, these answer options will be referred to by their commonly used English acronyms: respectively, DKO (“don’t know the object”), DKN (“don’t know the name”), and TOT (“tip of the tongue”). Participants were told that they had 7 s to look at each object and type in its name. The task took about 25 min.

In the *image agreement* task, participants were instructed that for each object they would first see its name (i.e., the modal name derived from the name agreement task, defined as the unique name that was produced by the largest number of participants in the name agreement task) on the 3-D screen in front of them for 4 s, after which they would see the corresponding 3-D object for 7 s. They were instructed to (passively—i.e., without saying it out loud) read the name of the object and imagine what an object corresponding to that name would normally look like. On a rating form, they then rated for each object the correspondence between their mental image and the presented 3-D object on a 5-point scale. A rating of 1 indicated *low agreement*, which meant a poor match to their mental image. A rating of 5 indicated *high agreement*, which meant a strong match to their mental image. For each object they were asked to circle *Geen Beeld* (“no image”) if they did not manage to form a mental image for an object, and *Ander Object* (“different object”) if they had a different object in mind than the one depicted. This task took about 35 min.

In the *familiarity* task, participants were instructed to look at each object and rate on a 5-point scale how familiar they were with the object. Familiarity was defined as the degree to which the participant usually comes in contact with the object or thinks about the concept. A rating of 1 indicated that the participant was *not familiar at all with the object*. A rating of 5 indicated that the participant was *very familiar with the object*. This task took about 25 min.

In the *visual complexity* task, participants were instructed to look at each object and rate on a 5-point scale how visually complex they found it. Complexity was defined as the amount of detail or the intricacy of the lines in each object. Color was not mentioned in the instructions. A rating of 1 indicated an *object with very few details*, and a rating of 5 indicated a *very detailed object*. This task took about 25 min.

### Apparatus

The CAVE system consisted of three screens (255 cm × 330 cm; VISCON GmbH, Neukirchen-Vluyn, Germany) that were arranged at right angles. Two projectors (F50, Barco N.V., Kortrijk, Belgium) illuminated each screen indirectly by means of a mirror behind the screen. For each screen, the two projectors showed two vertically displaced images that were overlapping in the middle of the screen. Thus, the complete display on each screen was visible only as a combined overlay of the two projections. Each object was presented on the screen facing the participants.

For optical tracking, infrared motion capture cameras (Bonita 10, Vicon Motion Systems Ltd, UK) and the Tracker 3 software (Vicon Motion Systems Ltd, UK) were used. Six cameras were positioned at the upper edges of the CAVE screens, and four cameras were placed at the bottom edges. All cameras were oriented toward the middle of the CAVE system. Optical head-tracking was accomplished by placing light reflectors on both sides of the VR glasses. Three spherical reflectors were connected on a plastic rack, and two such racks with a mirrored version of the given geometry were manually attached to both sides of the glasses. The reflectors worked as passive markers that can be detected by the infrared tracking system in the CAVE. The tracking system was trained to the specific geometric structure of the markers and detected the position and orientation of the glasses with an accuracy of 0.5 mm.

A control room was located behind the experimental room containing the CAVE setup. The experimenter could visually inspect the participant and the displays on the screens through a large window behind the participant. The four tasks were programmed and run using Python-based 3-D application software (Vizard, Floating Client 5.4; WorldViz LLC, Santa Barbara, CA).

## Results and discussion

Table 1 presents summary statistics for the following collected norms: the *H* statistic, the percentage of participants producing the modal name, image agreement, familiarity, and visual complexity. An Excel file available in the online database presents average measures for each individual 3-D object, in addition to the length (nchar; i.e., the number of letters) and lexical frequency of the modal name (SUBTLEXWF) derived from the online SUBTLEX-NL database (Keuleers, Brysbaert, & New, 2010), its English translation, and the object’s semantic category. Moreover, for the name agreement task, the numbers of DKO responses (1.33%), DKN responses (0.63%), and TOT responses (0.47%) are reported, as well as all nonmodal responses for each object. The average percentage of nonmodal responses obtained was 25.01%. Also, the number of times each object elicited the responses “no image” (0.34%) or “different object” (1.25%) in the image agreement task are provided online. On the basis of all these measures, researchers may select the 3-D objects that best fit the purpose of their study or application.

**Table 1:** Summary statistics for all elicited data

	*H*	%NA	IA	Fam	VC
*M*	1.05	74.99	3.91	3.20	2.69
*SD*	0.95	22.98	0.56	0.75	0.61
Median	0.81	80.95	3.98	3.12	2.69
Range	3.88	76.19	2.78	3.07	2.69
Min	0	23.81	2.17	1.76	1.45
Max	3.88	100	4.95	4.83	4.14
Q1	0.23	59.52	3.51	2.64	2.21
Q3	1.67	95.24	4.32	3.71	3.13
IQR	1.44	35.71	0.82	1.07	0.92
Skew	1.50	0.67	0.73	1.25	0.93

*H*, name agreement; %NA, percentage name agreement; IA, image agreement; Fam, familiarity; VC, visual complexity; Q1, 25th percentile; Q3, 75th percentile; IQR, interquartile range (Q3–Q1); skew = (Q3 – Median)/(Median – Q1), >1 indicates a positive skew

Table 2 presents the results of a Pearson correlation analysis between the different collected norms. Similar to other standardized databases, a significant negative correlation was observed between the *H* statistic and the percentage of participants producing the modal name (see Brodeur et al., 2010, for an overview). This indicates that the 3-D objects that elicited a larger number of different unique names also elicited a lower percentage of participants producing the modal name. The correlations between image agreement and the two measures of name agreement indicate that the 3-D objects that elicited a larger number of different labels evoked mental images that were more different from each actual 3-D object. Furthermore, more familiar 3-D objects had larger overlap with participants’ mental images, as indicated by the positive correlation between image agreement and familiarity. Finally, besides the commonly observed negative correlation between word length and word frequency, longer words were also rated as being visually more complex. This raises the interesting possibility that there may be an iconic relationship between the visual complexity of an object and the length of the verbal label it elicits (see Perniss & Vigliocco, 2014, for an overview of work on iconicity in human language).

**Table 2:** Correlation matrix for the collected norms

	*H*	%NA	IA	Fam	VC	nchar	WF
*H*	1.000						
%NA	–.959^**^	1.000					
IA	–.453^**^	.391^**^	1.000				
Fam	–.001	–.013	.200^*^	1.000			
VC	–.054	.072	–.042	.056	1.000		
nchar	.115	–.133	.145	–.086	.180^*^	1.000	
WF	.064	–.022	–.007	–.024	–.015	–.183^*^	1.000

*H*, name agreement; %NA, percentage name agreement; IA, image agreement; Fam, familiarity; VC, visual complexity; nchar, number of characters (i.e., word length); WF, word frequency. ^**^ *p* < .01 (two-tailed), ^*^ *p* < .05 (two-tailed)

Table 3 shows the mean values for name agreement, image agreement, familiarity, and visual complexity of the present 3-D object database and the corresponding mean values in three recently published databases that contain colored photographs (Adlington et al., 2009; Brodeur et al., 2010; Moreno-Martínez & Montoro, 2012). Furthermore, the corresponding mean values from the Snodgrass and Vanderwart (1980) black-and-white line drawing database are also given. The stimuli in the present 3-D object database and in the colored photograph databases on average elicited lower name agreement scores than did the line drawings by Snodgrass and Vanderwart. A lower overall name agreement facilitates the selection of stimuli that do not yield a ceiling effect in healthy participants in relatively simple tasks such as picture naming or object naming. This may be desirable when behavior in a healthy population is being compared to behavior in an impaired population (Adlington et al., 2009; Laws, 2005). Furthermore, comparison of the 25th (Q1) and 75th (Q3) percentile scores (Table 1) to the average scores for individual items in the online database will facilitate the selection of items from the extremes of the distribution.

**Table 3:** Overview of standardized measures in the present study, three recent colored photograph databases, and the canonical line drawings database by Snodgrass and Vanderwart (1980)

	*N*	*H*	%NA	IA	Fam	VC
Present study	147	1.05	74.99	3.91	3.20	2.69
Adlington et al. (2009)	147	1.11	67.61	n.e.	3.76	2.89
Brodeur et al. (2010)	480	1.65	64	3.9	4	2.4
Moreno-Martínez and Montoro (2012)	360	0.94	72	n.e.	3.56	2.55
Snodgrass and Vanderwart (1980)	260	0.56	n.e.	3.69	3.29	2.96

Mean scores for name agreement, image agreement, familiarity, and visual complexity are provided for comparison. *N*, number of objects/images; *H*, name agreement; %NA, percentage name agreement; Fam, familiarity; VC, visual complexity; n.e., not evaluated

The familiarity measure in the present study yielded a result numerically similar to that from the line drawing database by Snodgrass and Vanderwart (1980), which is slightly lower than the average familiarity ratings from the three databases with colored photographs. This difference may be due to the fact that both line drawings and 3-D objects are designed from scratch by a designer, whereas photographs of objects, by definition, represent objects more directly. Nevertheless, photographs and line drawings are typically 2-D abstractions of an actual 3-D real-world object. They represent an object, but they are not the represented object itself. In the case of 3-D VR research, however, a participant’s full immersion in a virtual world means that he or she should experience the 3-D objects as real objects. This difference also explains why certain semantic categories are not represented in the present database, though they are present in previous picture databases. Whereas traditional databases include, for instance, line drawings or (manipulated) photographs of individual body parts (Adlington et al., 2009; Duñabeitia et al., 2017; Moreno-Martínez & Montoro, 2012; Snodgrass & Vanderwart, 1980), no 3-D body parts are provided in the present database. Showing an individual body part in a 3-D virtual environment might decrease the participant’s experience of presence in the virtual world, since people usually do not come across individual, detached body parts in everyday life.

Numerically, the average image agreement and visual complexity of the 3-D objects in the present study are comparable to the norms for photographs and line drawings from the four other databases. The overall numerical similarity in image agreement suggests that, across the evaluated databases, participants on average agreed to a similar extent with the collected modal names. The overall similarity in average visual complexity scores suggests that the depicted objects in the present database, despite their 3-D nature, were not evaluated as being visually more complex than the stimuli in earlier databases. Note, however, that this might change if 2-D photographs of objects were directly compared to our 3-D objects in the same study with the same group of participants.

A more direct comparison of the present database to earlier databases was performed by running correlational analyses across items that elicited the same modal names across pairs of databases. Table 4 presents the results of these separate Pearson correlational analyses testing for correlations in name agreement, image agreement, familiarity, and visual complexity between the norms in the present study and those obtained using three previous stimulus databases. Items were included in a correlational analysis when the literal English translation of a 3-D object’s Dutch modal name corresponded to the English modal name in the database that was included in the analysis for comparison. The present database has 63 modal names in common with the photo database introduced by Brodeur et al. (2010). Moreover, it has 33 modal names in common with the color image database described in Moreno-Martínez and Montoro (2012). Fifty-two modal names from the present database were also elicited as modal names in the line-drawing database by Snodgrass and Vanderwart (1980). No correlational analyses were performed between the present database and the image database provided by Adlington et al. (2009), because only six modal names were the same across the two databases.

**Table 4:** Correlations (*r*) between ratings for 3-D objects from the present study, two recent colored photograph databases, and the canonical line drawings database by Snodgrass and Vanderwart (1980)

		Present study				
	*N*	*H*	%NA	IA	Fam	VC
Brodeur et al. (2010)	63					
*H*		.275^*^				
%NA			.211			
IA				.469^**^		
Fam					.516^**^	
VC						.569^**^
Moreno-Martínez and Montoro (2012)	33					
*H*		.160				
%NA			–.069			
IA				n.e.		
Fam					.670^**^	
VC						.584^**^
Snodgrass and Vanderwart (1980)	52					
*H*		.189				
%NA			.066			
IA				.420^**^		
Fam					.684^**^	
VC						.509^**^

*N*, number of items included in the analyses; *H*, name agreement; %NA, percentage name agreement; IA, image agreement; Fam, familiarity; VC, visual complexity. ^**^ *p* < .01 (two-tailed), ^*^ *p* < .05 (two-tailed)

Overall, significant correlations between the present database and previous databases in terms of name agreement were either absent (Moreno-Martínez & Montoro, 2012; Snodgrass & Vanderwart, 1980) or weak (Brodeur et al., 2010). Thus, although a modal name may be the same across studies, this does not imply that the name agreement for that specific item was also similar. This is not surprising, because different stimuli and different languages (Dutch, English, and Spanish) were involved in the different studies. Weak to moderate significant positive correlations were observed between the present database and previous databases in terms of image agreement. This suggests that, overall, certain modal names (e.g., “hammer”) elicit a highly stable mental image that is clearly represented by both picture stimuli and our 3-D object. Other modal names (e.g., “lamp”) may consistently elicit lower image agreement across different studies because there is more variance in the mental images each elicits (e.g., different types of lamps) across the participants within studies. Moderate to strong significant positive correlations were observed between the present database and the three earlier databases for both familiarity and visual complexity in all three comparisons (see Table 4).

The familiarity result indicates that, broadly speaking, objects that were normed as more or less familiar in the present study were also more or less familiar to the participants who provided norms in the earlier picture databases. This can be explained by the fact that the participants providing norms for the different databases have all lived in Western cultures in which they may encounter similar objects in their daily life. Some cultural differences in the familiarity of specific objects may exist, for example, in different culture-specific types of food (e.g., the typical Dutch pastry *tompouce* that was included in the present database, or the *crème caramel* in Moreno-Martínez & Montoro, 2012). Such items were, however, by definition not included in these analyses, because they were present in only one of the databases.

The positive correlations in terms of visual complexity suggest that the objects depicted as visually more or less complex in the earlier databases were also designed and rated as being visually more or less complex in the present database. This overlap is explained by the inherent degree of visual complexity present in objects in everyday life, which is consequently represented as such in line drawings, pictures, and 3-D objects based on these real-world objects.

All in all, the comparisons of the present 3-D object database to four previous databases confirm the validity of the present set of 3-D objects. On the basis of these results, the present standardized 3-D object database sets the stage for better comparability of scientific findings that can result from the use of immersive VR and augmented reality settings within and across research labs and participant groups.

### Conclusion

This study has introduced the first standardized set of 3-D objects for VR and augmented reality research and applications. The objects are freely available and can be selected as a function of the aim of a specific study or application, on the basis of the provided norms for name agreement, image agreement, familiarity, visual complexity, and the lexical characteristics of the object’s modal name. The 3-D objects can be adapted in size, color, texture, and visual complexity to fit the purposes of individual studies and applications. Note, however, that the collected norms are representative only of the 3-D objects as they are currently presented in the online database. Modifying, for instance, an object’s texture or color might change any of the collected norms. The 3-D objects can be used further for educational purposes as well as for testing patient populations in 3-D virtual environments. Researchers performing experiments in languages other than Dutch are invited to standardize the current set of 3-D objects for their local language and to expand the database by adding more objects. Sharing standardized 3-D objects across different labs will move VR research forward more quickly.
